# Supplementary material for: A Non-Specific Phytohormone Regulatory Network in Saccharina japonica Coordinates Growth and Environmental Adaptation
Source: Plants (Basel). 2025 Jun 13;14(12):1821. doi: 10.3390/plants14121821 (PMC12196923; doi:10.3390/plants14121821)
Supplement: Supplementary file 1 [file plants-14-01821-s001.zip › Figures S1-S12.pdf]

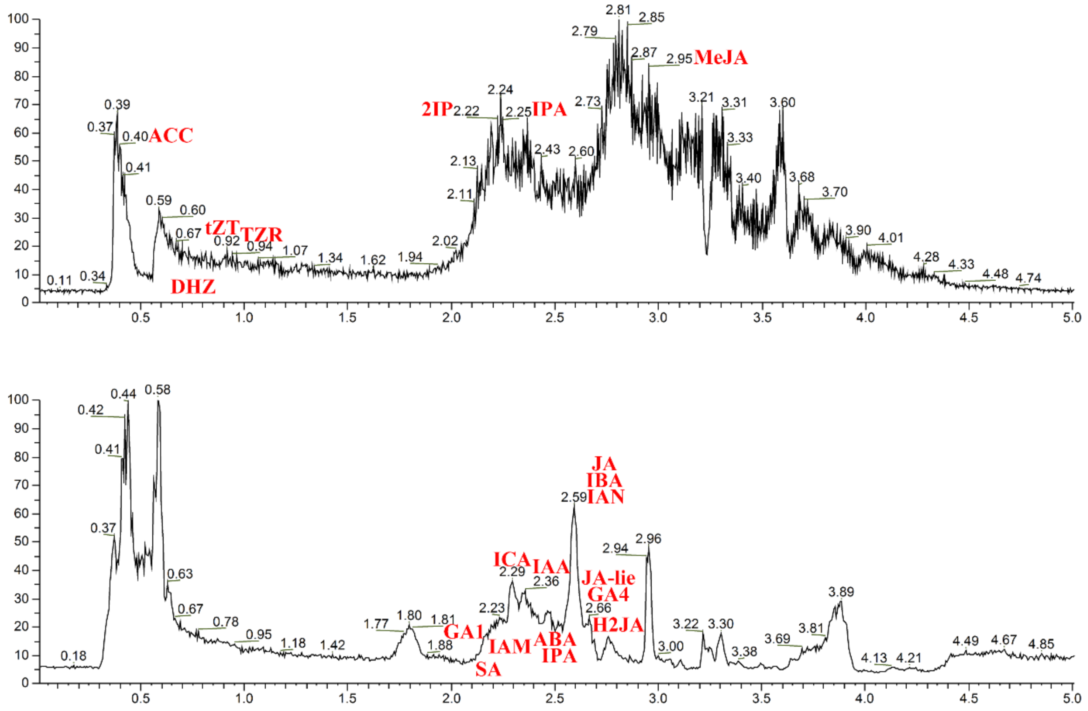

Figure S1. HPLC-MS/MS total ion chromatograms of extracts from different parts of *S. japonica*. (A) Positive ion mode of extracts from different parts of *S. japonica*. (B) Negative ion mode of extracts from different parts of *S. japonica*. ABA: abscisic acid. tZT: trans-zeatin. TZR: trans-zeatin-riboside. 2IP: N6-(delta2-isopentenyl) adenine. IPA: N6-(delta2-isopentenyl) adenosine. DHZ: dihydrozeatin. GA3: gibberellic A3. GA4: gibberellin A4. MeJA: methyl jasmonate. H2JA: dihydrojasmonic acid. JA: jasmonic acid. JA-lie: jasmonic acid-isoleucine. ACC: aminocyclopropane carboxylic acid. SA: salicylic acid. IAA: indole-3-acetic acid. IBA: 3-indolebutyric acid. ICA: 3-indolecarboxylic acid. IPA: 3-indolepropionic acid. IAN: 3-indoleacetonitrile. IAM: 3-indoleacetamide.

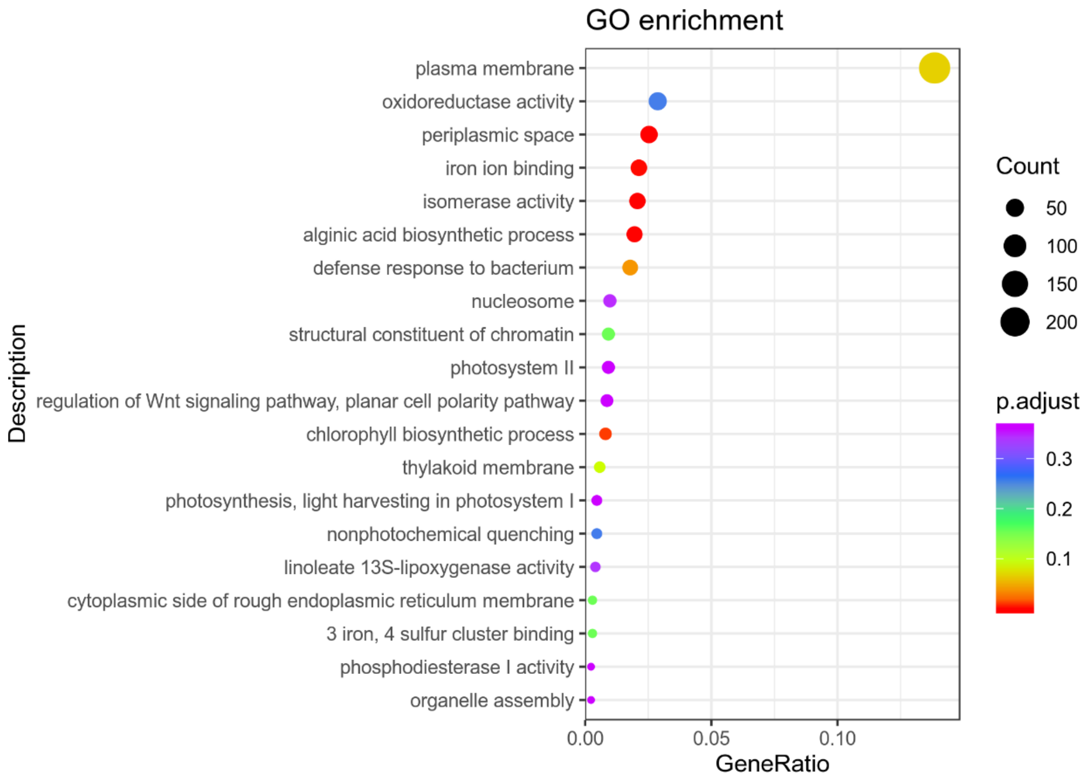

Figure S2. GO enrichment bubble maps of DEGs for R vs. P.

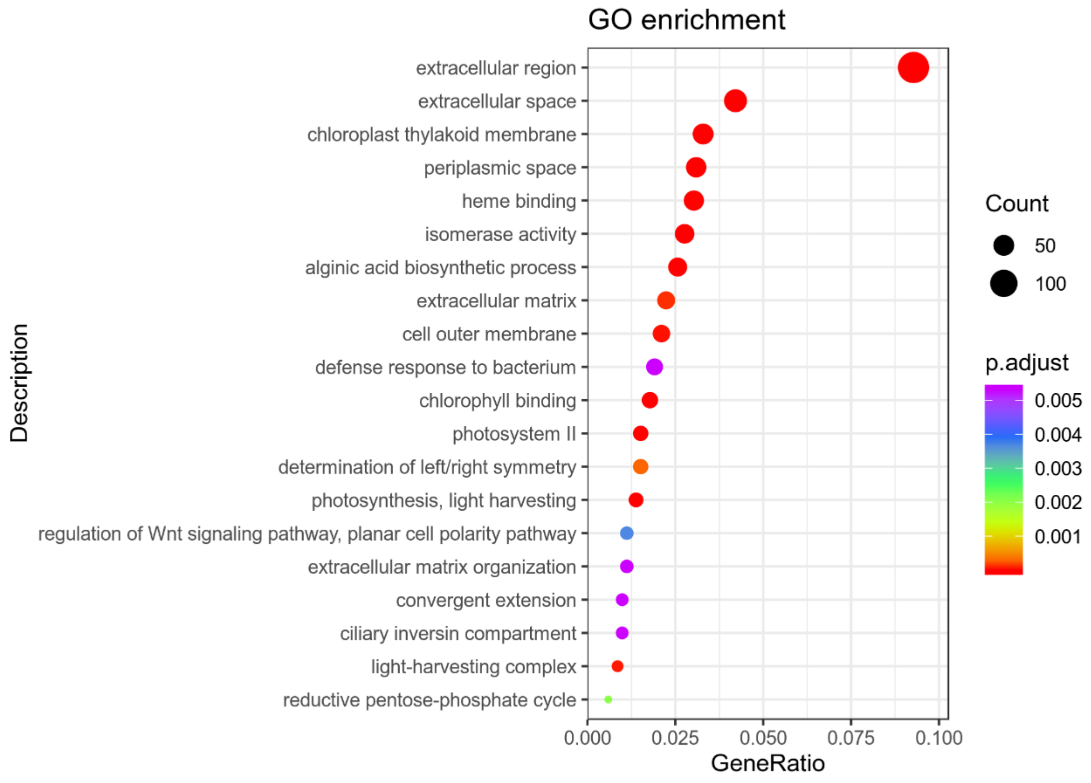

Figure S3. GO enrichment bubble maps of DEGs for R vs. B.

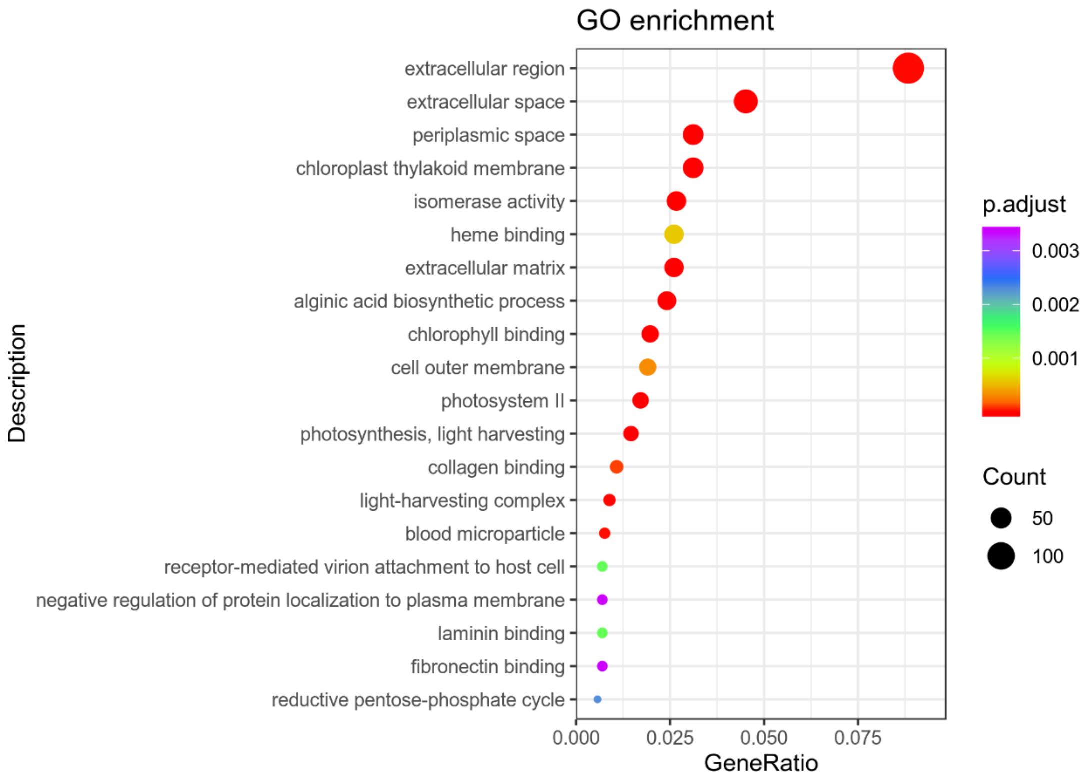

Figure S4. GO enrichment bubble maps of DEGs for R vs. M.

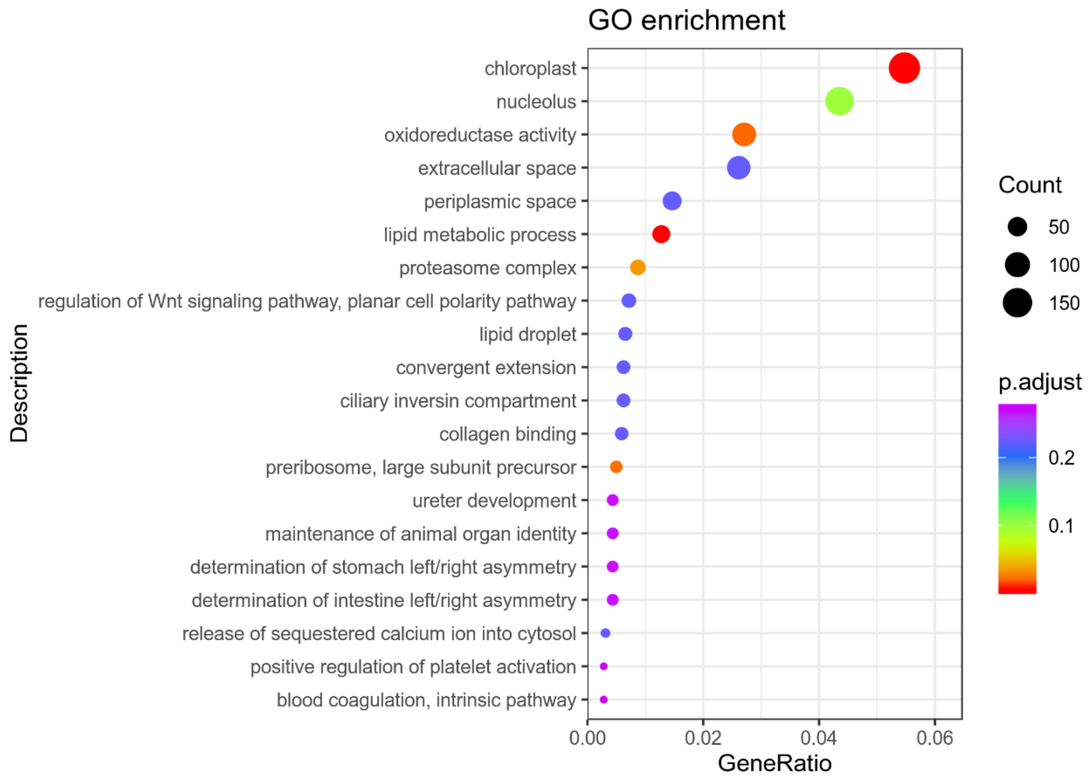

Figure S5. GO enrichment bubble maps of DEGs for R vs. T.

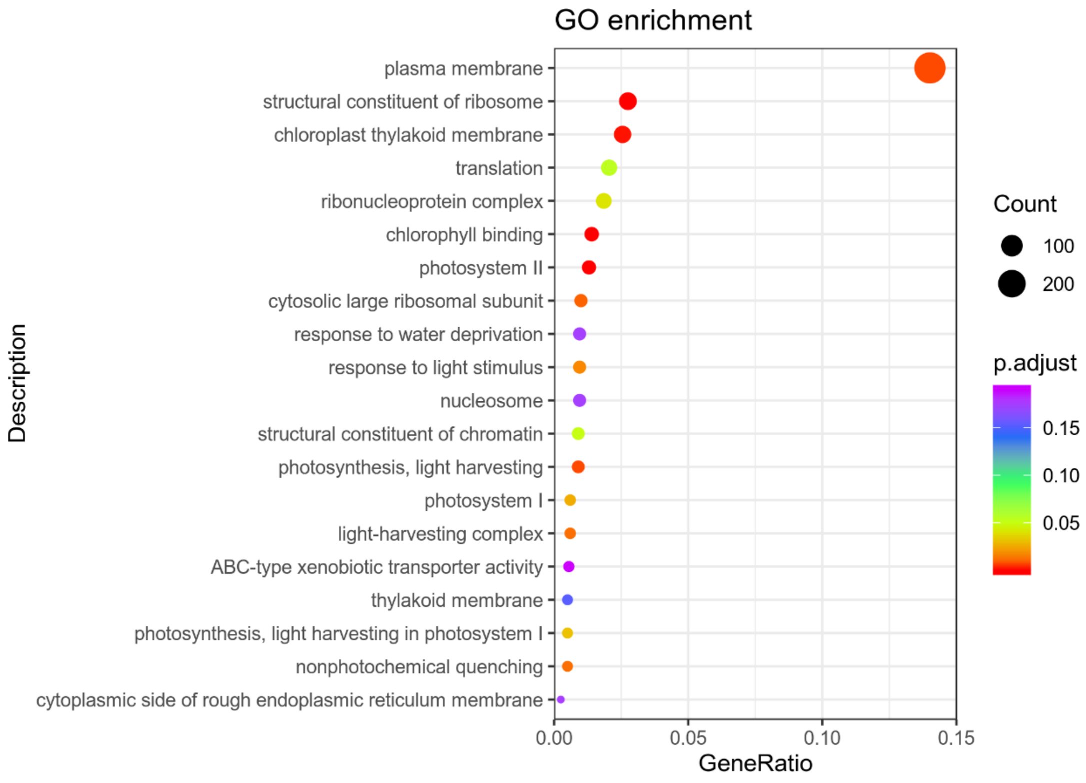

Figure S6. GO enrichment bubble maps of DEGs for P vs. B.

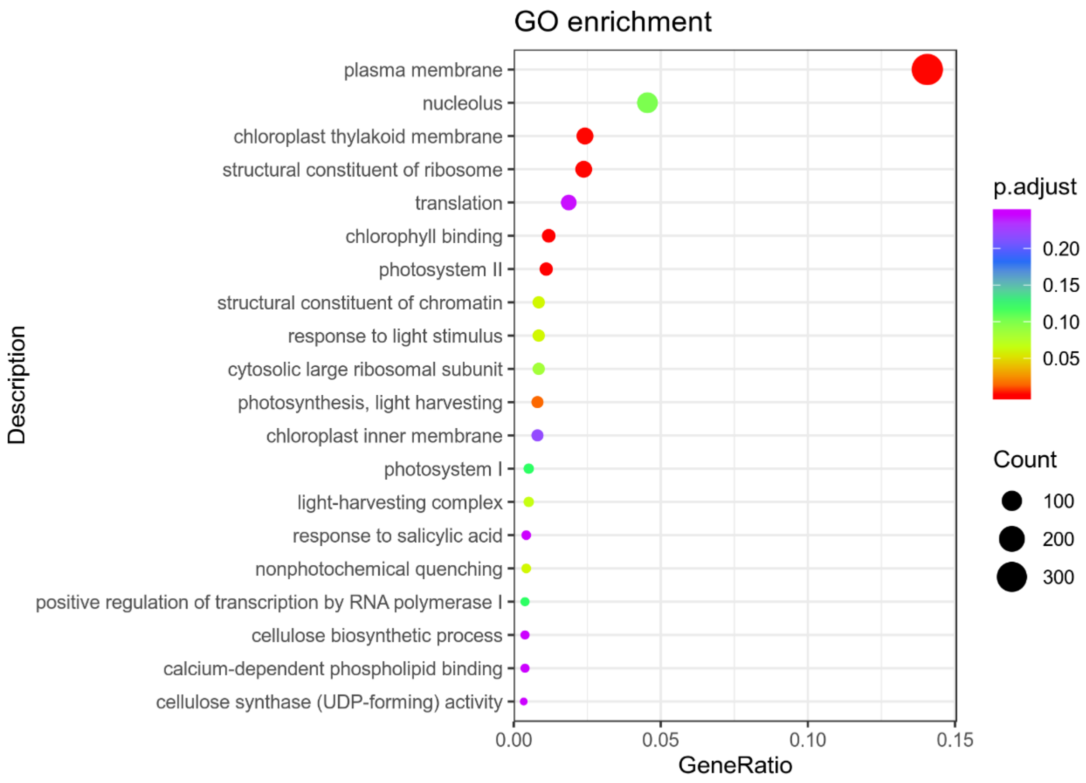

Figure S7. GO enrichment bubble maps of DEGs for P vs. M.

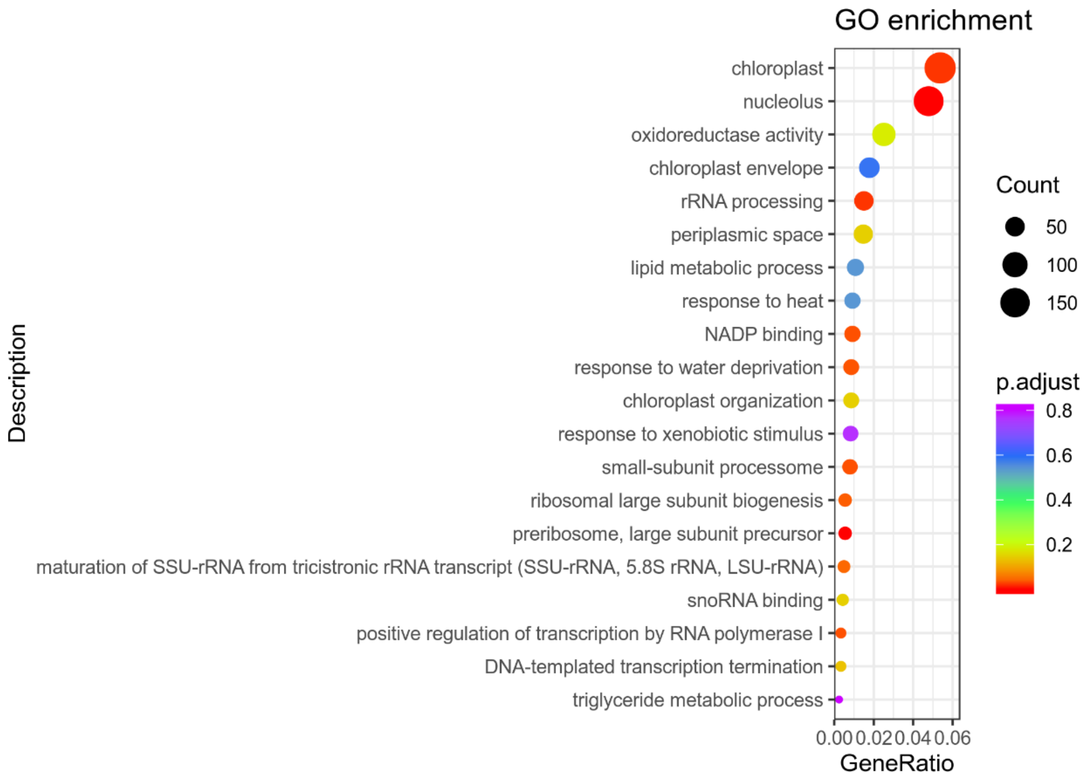

Figure S8. GO enrichment bubble maps of DEGs for P vs. T.

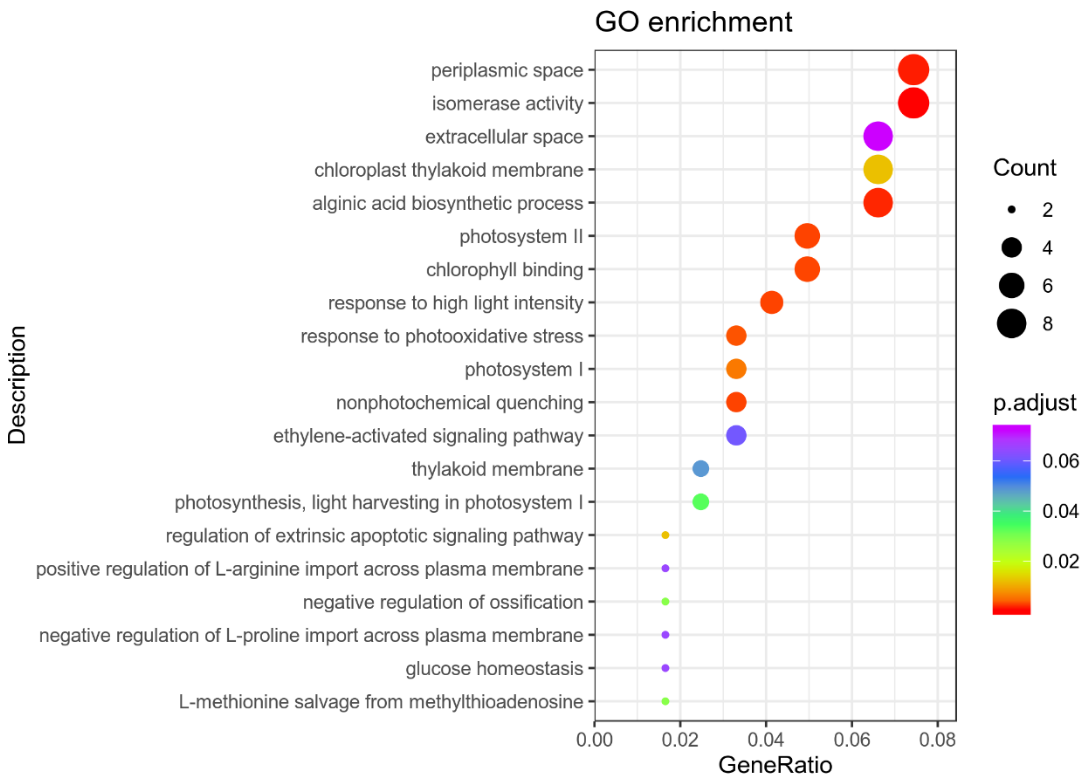

Figure S9. GO enrichment bubble maps of DEGs for B vs. M.

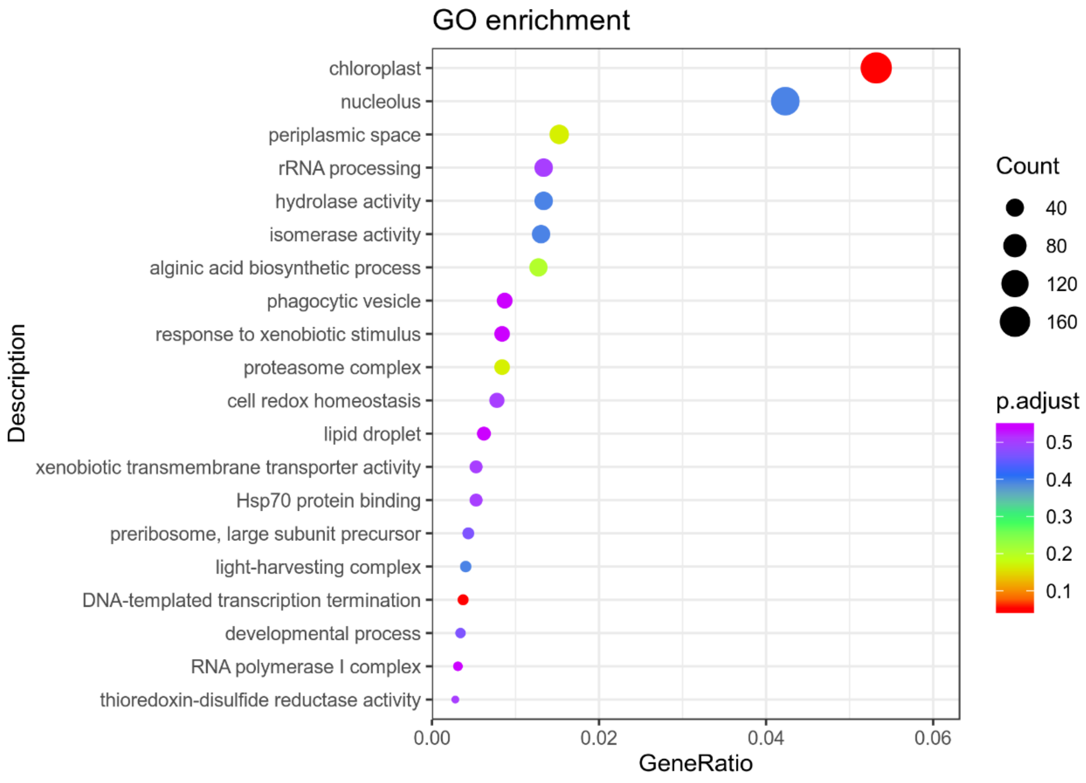

Figure S10. GO enrichment bubble maps of DEGs for B vs. T.

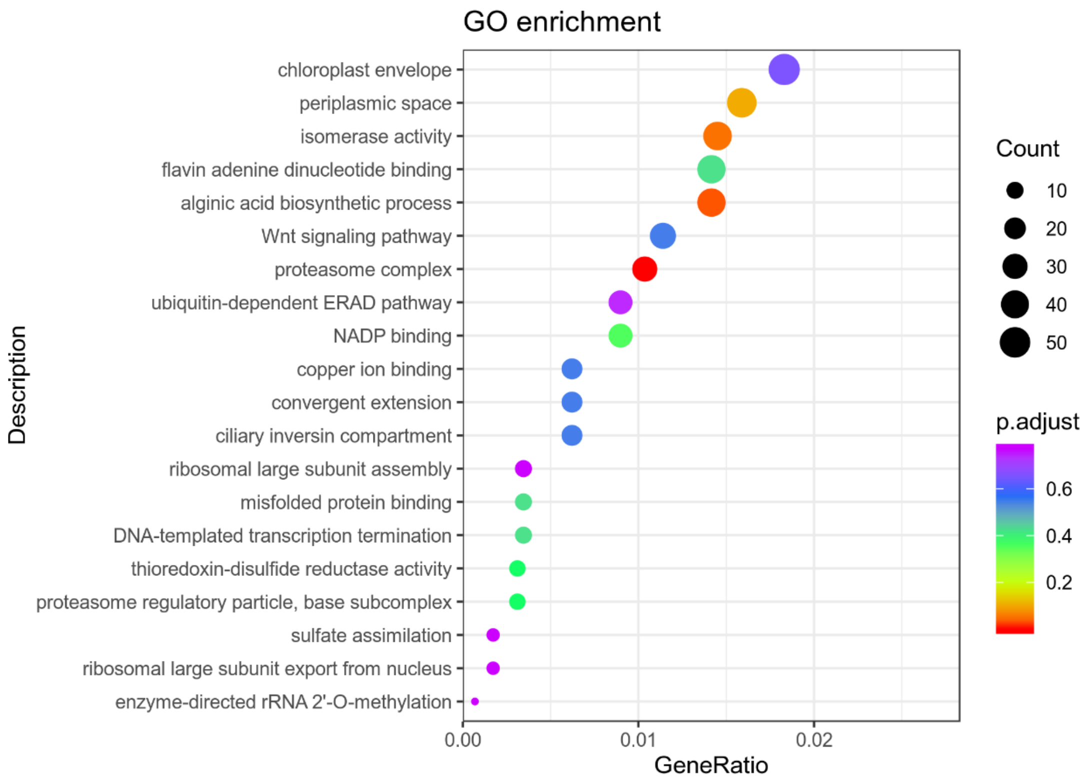

Figure S11. GO enrichment bubble maps of DEGs for M vs. T.

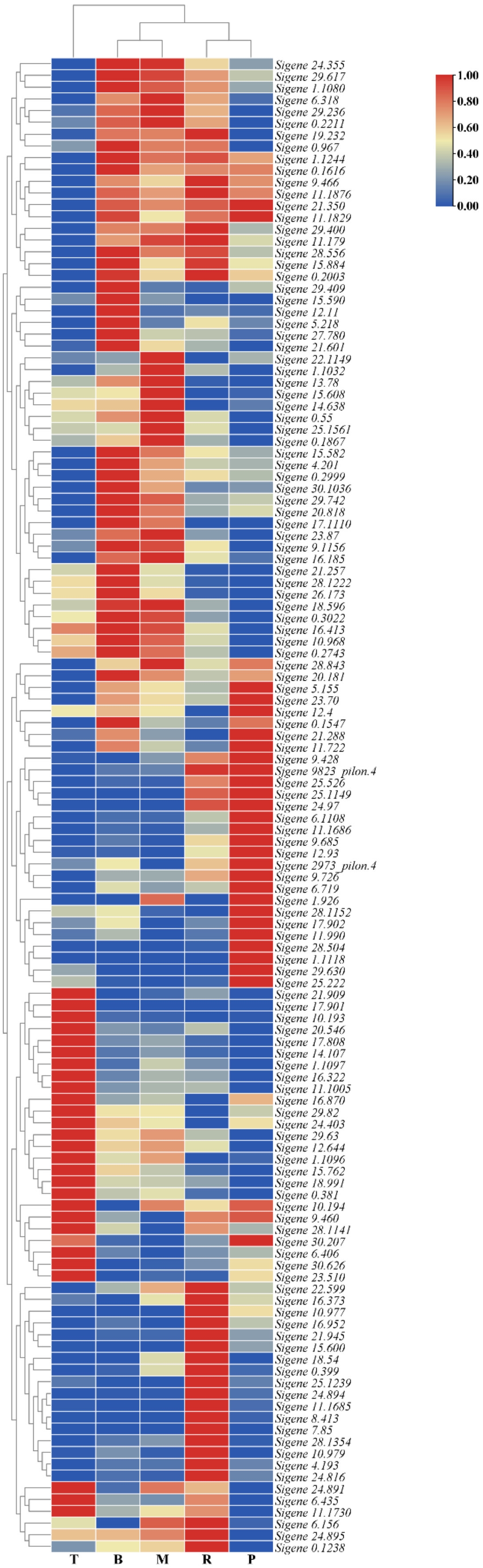

Figure S12. Expression of phytohormone biosynthesis related genes in R, P, B, M, and T of *S. japonica*.
